# miR-330–5p in Small Extracellular Vesicles Derived From Plastrum testudinis-Preconditioned Bone Mesenchymal Stem Cells Attenuates Osteogenesis by Modulating Wnt/β-Catenin Signaling

**DOI:** 10.3389/fmolb.2021.679345

**Published:** 2021-08-09

**Authors:** Xiaoyun Li, Yan Cui, Qing Lin, Panpan Wang, Rumeng Chen, Xiaofeng Zhu, Li Yang, Ronghua Zhang

**Affiliations:** ^1^ College of Pharmacy, Jinan University, Guangzhou, China; ^2^ College of Traditional Chinese Medicine, Jinan University, Guangzhou, China; ^3^ The First Affiliated Hospital of Jinan University, Guangzhou, China; ^4^ Cancer Research Institution, Jinan University, Guangzhou, China

**Keywords:** small extracellular vesicles, miR-330-5p, bone mesenchymal stem cell, osteogenesis, plastrum testudinis

## Abstract

The bone microenvironment is crucial for the growth and development of different types of osteocytes. Small extracellular vesicles (sEVs) secreted by bone mesenchymal stem cells are delivered to target cells where their contents regulate biological functions. Here, we evaluated the osteogenic effects and mechanism of sEVs derived from *Plastrum testudinis*-preconditioned bone mesenchymal stem cells (PT-sEV). The osteogenic effects of PT-sEV were evaluated by the differentiation of osteoblasts and the alternation of bone quality and quantity in ovariectomized rats. The specific mechanism was explored by high-throughput sequencing and verified by transfection with the corresponding miRNA mimic and inhibitor. RNA-sequence identified a unique enrichment of a set of miRNAs in PT-sEV compared with sEVs derived from untreated BMSCs. Overexpression or inhibition *in vitro* indicated that the osteogenic inducing potential of sEVs was mainly attributable to miR-330-5p, one of the most dramatically downregulated miRNAs in the PT-sEV fraction. Dual luciferase reporter assays showed that miR-330-5p negatively regulated osteogenesis by directly binding to the 3′ untranslated region of *Tnc*. Additional experiments showed that *Tnc* regulated Wnt/β-catenin signaling, and rescue experiment showed that miR-330-5p could restore β-catenin expression; additionally, animal experiments indicated that Wnt signaling was inactivated in the ovariectomized rats. These data demonstrated the regenerative potential of PT-sEV, which induced osteogenic differentiation of pre-osteoblasts, leading to bone formation. This process was achieved by delivering miR-330-5p, which regulated *Tnc* to control Wnt/β-catenin signaling.

## Introduction

The balance of bone metabolism is closely maintained by the rates of bone formation and resorption (Appelman-Dijkstra and Papapoulos, 2015). Previous studies have indicated that altering the bone microenvironment could directly influence bone formation and resorption (Ma and Yu, 2020). Bone mesenchymal stem cells (BMSCs) are a type of multi-potent stem cells that are located in the bone marrow, where they secrete various factors and can differentiate into cell of an appropriate phenotype, making it promising therapeutic candidates for osteoporosis treatments. Previous studies have shown that the therapeutic potential of MSCs is mostly attributable to paracrine mechanisms, particularly MSCs-derived small extracellular vesicles (sEVs) (Li et al., 2018).

It is well accepted that sEVs plays indispensable roles in modulating major cellular processes by delivering cargo such as nucleic acids, proteins, and lipids to receiving cells and are associated with fewer safety concerns (Yue et al., 2020). When the contents of sEVs are transferred to a target cell they can regulate its function. miRNAs are widely distributed in sEVs and are a form of non-coding RNA that regulate gene activities by binding to the 3′ untranslated region (UTR) of target gene. For example, sEVs derived from bone marrow stromal cells contain miR-146a, which regulates osteogenesis and angiogenesis (Liu et al., 2021). miR-214-3p derived from osteoclast sEVs targets osteoblasts and inhibits bone formation (Li et al., 2016). Furthermore, the status of the donor cells might determine the biological functions of their generated sEVs (Su et al., 2017). Therefore, we hypothesized that sEVs derived from BMSCs could influence osteoblast activity and bone formation.

*Plastrum testudinis* (PT) is a traditional Chinese medicine that is the dried plastron and carapace of the tortoize *Chinemys reevesii* (Chinese Pharmacopoeia Com, 2020). Researches have indicated various biological activities, including promoting collagen synthesis, inducing BMSCs differentiation, and anti-osteoporosis Chen et al. (2007); Wang et al. (2012) found that PT extracted from petroleum ether or ethyl acetate promoted BMSCs proliferation. Ren et al. (2017) showed that PT stimulates bone mass, microarchitecture, and the expression of bone turnover markers in glucocorticoid-induced osteoporosis. We wondered if sEVs derived from PT-preconditioned BMSCs (PT-sEV) could stimulate bone formation, and then addressed the underlying mechanism.

In this study, we first investigated the osteogenic differentiation of BMSCs with PT intervention, and then collected PT-sEV to evaluate their osteogenic effect on pre-osteoblasts and ovariectomized rats. We also analyzed the miRNA profile of PT-sEV to further investigate if the specific mechanism was miRNA-mediated. Our findings proved the potential of PT-sEV on osteogenesis and detailed the specific mechanism, which may be highly beneficial for the future development of novel bone regeneration strategies.

## Materials and Methods

### Preparation and Analysis of Water-Extracted PT

PT was twice extracted by decoction in distilled water (2 h each time), and then concentrated via rotary evaporation to a final concentration of 0.1 g/ml. After cooling the extracts to room temperature, PT was stored at −20°C under dry, airproof, and non-polluted conditions until further use. Qualitative amino acid analyses of the PT extracts were performed using a Hitachi L-8900 automatic amino acid analyzer (Tokyo, Japan).

### Cells Culture and Treatment

Rat BMSCs were purchased from Cyagen Bioscience (Guangzhou, China). Pre-osteoblasts were obtained from 3 day-old rats, and P3-P8 primary cells were used in the following experiments; the pre-osteoblasts extraction procedures were performed in accordance with a previous study (Torricelli et al., 2003). Both cell types were cultured in α-MEM (Gibco, Grand Island, NY, United States) containing 10% fetal bovine serum (FBS500-S, Ausgenex Pty, Australia) at 37°C with 5% CO_2_.

For the co-culture system, pre-osteoblasts were seeded into a 6-well plate at a density of 5 × 10^3^ cells/well and BMSCs were seeded onto a six-well transwell filter at a density of 5 × 10^3^ cells/well (0.4 μm pore size), which were incubated for 6 h upon cell adherence. The co-cultures were then treated with/without GW4869 (It is a commonly used pharmacological agent, which inhibits sEVs generation) (D1692; Sigma-Aldrich, United States) or PT for 48 h, then total pre-osteoblasts RNA was extracted and analyzed by quantitative (q)PCR.

### Isolation, Characterization, and Uptake of sEVs

BMSCs were plated in 10 cm culture dishes at a density of 5 × 10^5^ cells/dish, and then the culture medium was replaced with exosome-free serum medium with/without 1 μg/ml PT for 48 h when the cells reached 80% confluence. The culture medium was then collected and centrifuged at 300 *×g* for 10 min, 3,000 *×g* for 10 min, 10,000 *×g* for 30 min, and then 100,000 *×g* for 1 h twice, each time discarding the supernatant and resuspending the pellet with PBS. The final pellets were stored at −80°C until further use. The collected samples included two groups: 1) sEVs derived from untreated BMSCs (Un-sEVs); and 2) PT-sEV.

The concentration of sEVs was evaluated by BCA protein assay kits (23,225, Thermo Fisher Scientific, United States), and sEVs were characterized via electron microscopy, a nanoparticle tracking analyzer (NTA), and Western blot, which was performed with anti-CD9 (ab92726, abcam, United States), anti-Alix (NBP1-90201, Novusbio, United States), and anti-Tsg101 (NBP1-80659, Novusbio, United States) antibodies.

### Cell Counting Kit 8

BMSCs were seeded into 96-well plates at a density of 5 × 10^3^ cells/well, then treated with different concentrations of PT (10^−3^, 10^−2^, 10^−1^, and 1, 10 μg/ml). After culturing for 24 or 48 h, cell proliferation was measured using a CCK8 according to the manufacturer’s instructions (CK04, Dojindo, Japan). Briefly, the culture medium was replaced with 100 μl α-MEM containing 10 μl CCK8, and the plates were incubated for 1 h at 37°C. Absorbance was measured at 450 nm using a multi-well spectrophotometer (BioTek, Synergy H4).

### Alkaline Phosphatase Assay

BMSCs were seeded into six-well plates at a density of 2 × 10^3^ cells/well, and treated with different concentrations of PT, as described in 2.4. Cells were treated with PT for 3 and 7 days, and ALP activity assays were completed using ALP kits according to the manufacturer's instructions (A059-2-2, Nanjing Jiancheng, China). Absorbance was measured at 520 nm using a multi-well spectrophotometer (BioTek, Synergy H4).

### Alizarin Red S Staining

BMSCs were seed into the 60 cm culture dish at the density of 1 × 10^4^ with different treatment. Briefly, the culture medium was replaced every two days, then on day 28 of culture, the medium was discarded, and the cells were fixed in 75% ethanol for 10 min, then washed with PBS, and stained with 0.1% alizarin red S (G1450, Solarbio, China). After staining for approximately 5 min at room temperature, the wells were rinsed to remove excess dye and images were captured by microscopy (Zesis, AXIO), each group captured three pictures and quantified by ImageJ (NIH, Bethesda, MD, United States).

### Animals and Treatments

Female 2-month-old Sprague–Dawley rats underwent bilateral ovariectomies according to a previous study (Liu et al., 2018). Four weeks post-surgery, the ovariectomized (OVX) rats were randomly divided into three groups (*n* = 8 per group): a sham control group (Sham; sham surgery with phosphate buffer saline tail vein injections), an ovariectomy control group (OVX; ovariectomy surgery with phosphate buffer saline [PBS] treatment), and the sEVs intervention group (PT-sEV; ovariectomy surgery with PT-sEV). For the PT-sEV group, tail vein injections included PT-sEV (100 ng/100 g/d) for 10 weeks; the other two groups were treated with the same volume of PBS. At the timepoint, the rats were sacrificed and samples were collected for further use.

### Bone Mineral Density and Micro Computed Tomography Analyses

The BMD of the femur was analyzed by dual-energy X-ray absorptiometry (DXA) (General Electric Company, Healthcare), which used specific software to assess bone density in small animals. The results are analyzed by lunar_iDXA software and presented in g/cm^2^.

The trabecular micro-architecture of the femur was evaluated by micro-CT scan (SkyScan 1,176, Kontich, Belgium). The parameters were set as follows: 50 kV voltage, 400 μA current, and 8.88 μm per pixel resolution. A refined volume of 0.5 mm below the growth plate and 1 mm in height was chosen for further qualitative and quantitative analyses. Then the bone volume/tissue volume (BV/TV), trabecular thickness (Tb.Th), trabecular separation (Th.Sp), and trabecular number (Tb.N), Trabecular Bone Pattern factor (TBpf), as well three-dimensional images were obtained for visualization and display.

### Hematoxylin and Eosin Staining and Immunochemistry (IHC)

Femur and lumbar vertebrae were fixed with 4% paraformaldehyde, decalcified with 18% EDTA, dehydrated, paraffin embedded, sectioned at 10 μm thickness onto glass slides, and then stained with H and E. These procedures were conducted according to previously published methods (Chinese Pharmacopoeia Com, 2020). All the images were captured by microscope (Zesis, AXIO).

The paraffin sections were also used for IHC experiments. The 10 μm thick sections were prepared, stained, counterstained, dehydrated, hyalinized, and mounted; the antibody dilution for IHC was 1: 100. All the images were captured by microscope (Zesis, AXIO)

### Western Blotting and Immunofluorescence

Total protein was extracted by RIPA lysis (P0013B, Beyotime, China) and the concentration was measured by BCA kits. Total protein was separated by SDS-PAGE electrophoresis and transferred to PVDF membranes, which were incubated with the following primary antibodies at a 1:1,000 dilution and 4°C overnight: anti-ALP (GTX42809, GeneTex, United States of America), anti-COL1A1 (E8F4L, Cell signaling technology, United States), anti-RUNX2 (D1H7, Cell signaling technology, United States of America), and anti-BMP-2 (ab225898, abcam, United States). The following day, the membranes were incubated with anti-rabbit IgG secondary antibody (1: 3,000, 7074P2, Cell signaling technology, United States). Immunoreactive bands were visualized with an ultrasignal chemiluminescent reagent (4A Biotech, Beijing, China). The results were quantified using ImageJ (NIH, Bethesda, MD, United States).

Immunofluorescence was conducted according to the manufacturer’s instructions (P0183, Beyotime, China). Briefly, the culture medium was discarded and the cells were fixed with 95% ethanol. The samples were then washed three times for 15 min with TBSTx and blocked with 5% BSA. BMP-2 antibodies (ab225898, abcam, United States) were then incubated with the cells overnight at 4°C. Thereafter, 4’,6-Diamidino-2-phenylindole (C1005, Beyotime, China) was used to stain nuclei before capturing images. Images were captured by a fluorescence microscope (Zesis, AXIO). Each group was captured three pictures and the results were calculated using ImageJ (NIH, Bethesda, MD, United States).

### Enzyme-Linked Immunosorbent Assay

Molecular markers of bone turnover including bone gla protein (BGP), estradiol, parathyroid hormone (PTH), and C-terminal telopeptide (CTX) were measured using commercial ELISA kits from eLabscience (Houston, TX, United States). Calcium and phosphorus levels were measured by an automatic biochemical analyzer (Hitachi 7,020).

### RNA-Sequencing and Real-Time qPCR

Total miRNA was extracted using miRNA mini kits (217,004, Qiagen, Germany); the quality and quantity of miRNA were evaluated using a Nanodrop 2000 (Thermo Fisher Scientific, United States), as well a RNA integrity analysis was carried out by an agarose gel electrophoresis. Then miRNA was transcribed into cDNA and amplified following the manufacturer’s instructions (638,313, Takara, Japan). Raw data from small miRNA were obtained using illumine. The fold change and *p*-value were calculated for each miRNA, and the data were filtered with the standards: fold change ≥2.0 and FDR <0.05. Target genes of the identified miRNAs were predicted using TargetScan and miRbase.

Total RNA was extracted using TRIzol reagent (15,596,026, Thermo Fisher Scientific, United States). Then mRNA were transcribed into cDNA and amplified following the manufacturer’s instructions (820A; Takara, Japan). Relative gene expression was calculated using formula 2^−△△ct^, and raw data were standardized to GAPDH. The related primers are listed in Supplementary Material S1.

### Transfection of miRNA Mimic or Inhibitor and Luciferase Reporters

BMSCs or pre-osteoblasts were transfected with 50 nM of miR-330-5p mimic or 100 nM of the corresponding inhibitor. Briefly, upon reaching 70% confluence, the cells were incubated with 500 μl of transfection mix (Lipofectamine 3,000; Invitrogen, L3000008, United States) with mimic or inhibitor (miR20005, Ruibo, China) at 37°C for 24 h. Transfection efficiencies were evaluated by qPCR.

The 3’UTR of Tnc was amplified and cloned into the firefly reporter vector (Genematrix, Inc. Seongnam, Korea). A mutant version of the Tnc 3’UTR reporter plasmid was also constructed. The Dual-Glo luciferase assay system (E1910; Promega, United States) was used to evaluate luciferase activity 48 h post-transfection. Normalized firefly luciferase activity data were compared between the different groups.

### Statistical Analysis

All data are presented as mean ± standard deviation or replicate values, and were analyzed using GraphPad Prism 8.0 (GraphPad Software, Inc. San Diego, CA, United States). Each experiment was repeated three times. Multiple-group comparisons were evaluated by one-way analysis of variance (ANOVA) with the Newman–Keuls test. *p* < 0.05 was considered a significant difference.

## Results

### PT Stimulated Osteogenic Differentiation of BMSCs

**FIGURE 1:**
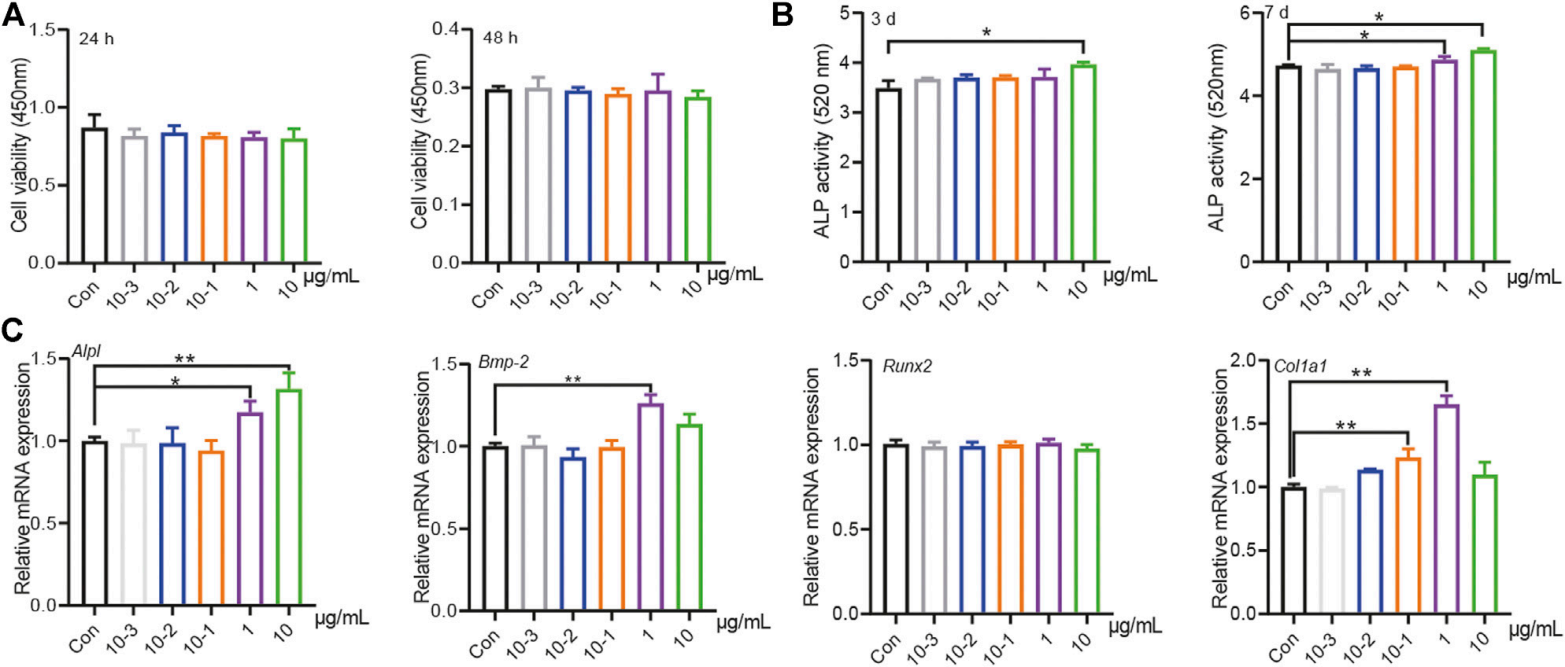
PT stimulated osteogenic differentiation of BMSCs. **(A)** The effects of different concentration (10^–3^, 10^–2^, 10^–1^, 1, 10 μg/ml) PT on proliferation in BMSCs at 24 or 48 h, the raw data represented the mean ± SD; **(B)** The effects of different concentration PT on the secretion of ALP in BMSCs at 3rd or seventh day, the raw data represented the mean ± SD; **(C)** The effects of different concentration PT on mRNA of osteogenic related factors in BMSCs, the normalized data represented the mean ± SD. *p < 0.05 *vs* Con, **p < 0.01 *vs* Con, analyses were done twice and obtained comparable results.

Qualitative and relative quantitative analyze of the amino acid constituents of water-extracted PT was performed by an automatic amino acid analyzer, including detection of indispensable amino acids and essential amino acids (Table 1). We then evaluated the effects of different PT concentrations on the proliferation and osteogenic differentiation of BMSCs. The results showed that there was no significant difference among different PT concentrations on the proliferation of BMSCs at 24 or 48 h (Figure 1A). The alkaline phosphatase (ALP) assay was used to analyze osteogenic differentiation, and the results showed that 1 and 10 μg/ml PT significantly promoted ALP secretion on day 3. ALP levels were also increased on day 7 with 10^−1^, 1, and 10 μg/ml PT (Figure 1B). The qPCR results revealed that 1 μg/ml PT significantly increased mRNA levels of *Col1a1, Alpl, and Bmp-2*; 10 μg/ml PT increased the expression of *Alpl* and *Bmp-2* mRNA (Figure 1C). Combining these results, we chose 1 μg/ml PT for subsequent experiments.

**TABLE 1:** The prime sequences.

Gene name	5’-3’ sequence	
***Bmp-2***	Forward	GCC​ATC​GAG​GAA​CTT​TCA​GA
	Resverse	TGT​TCC​CGA​AAA​ATC​TGG​AG
***Alpl***	Forward	GAC​AAG​AAG​CCC​TTC​ACA​GC
	Resverse	ACT​GGG​CCT​GGT​AGT​TGT​TG
***Col1a1***	Forward	ACG​TCC​TGG​TGA​AGT​TGG​TC
	Resverse	TCC​AGC​AAT​ACC​CTG​AGG​TC
***Runx2***	Forward	AAC​AGC​AGC​AGC​AGC​AGC​AG
	Resverse	GCA​CGG​AGC​ACA​GGA​AGT​TGG
***β-catenin***	Forward	GAA​AAT​GCT​TGG​GTC​GCC​AG
	Resverse	ATG​GCA​GGC​TCG​GTA​ATG​TC
***Tcf***	Forward	TAC​AGG​GTT​GCC​ACC​AGA​GT
	Resverse	CTG​TGC​CTG​CTG​AGA​GTG​AA
***Wnt3a***	Forward	TGG​TGG​TGG​TGG​TGG​CAG​AG
	Resverse	CAC​AGC​CAA​GGA​CCA​GAG​AAG​AAC
***Lrp5***	Forward	GGA​CAT​CGA​GTT​TGG​TGG​GA
	Resverse	GTT​GTT​GTG​GCG​GTT​CAT​GG
***Lef***	Forward	CCC​ATC​TTC​ACT​TTC​AGG​GGA​C
	Resverse	TAG​CGT​ACA​CTC​GGC​TAC​GA
***Gapdh***	Forward	GAC​ATG​CCG​CCT​GGA​GAA​AC
	Resverse	AGC​CCA​GGA​TGC​CCT​TTA​GT

### The Osteogenic Effects of PT-sEV on Pre-osteoblasts

**FIGURE 2:**
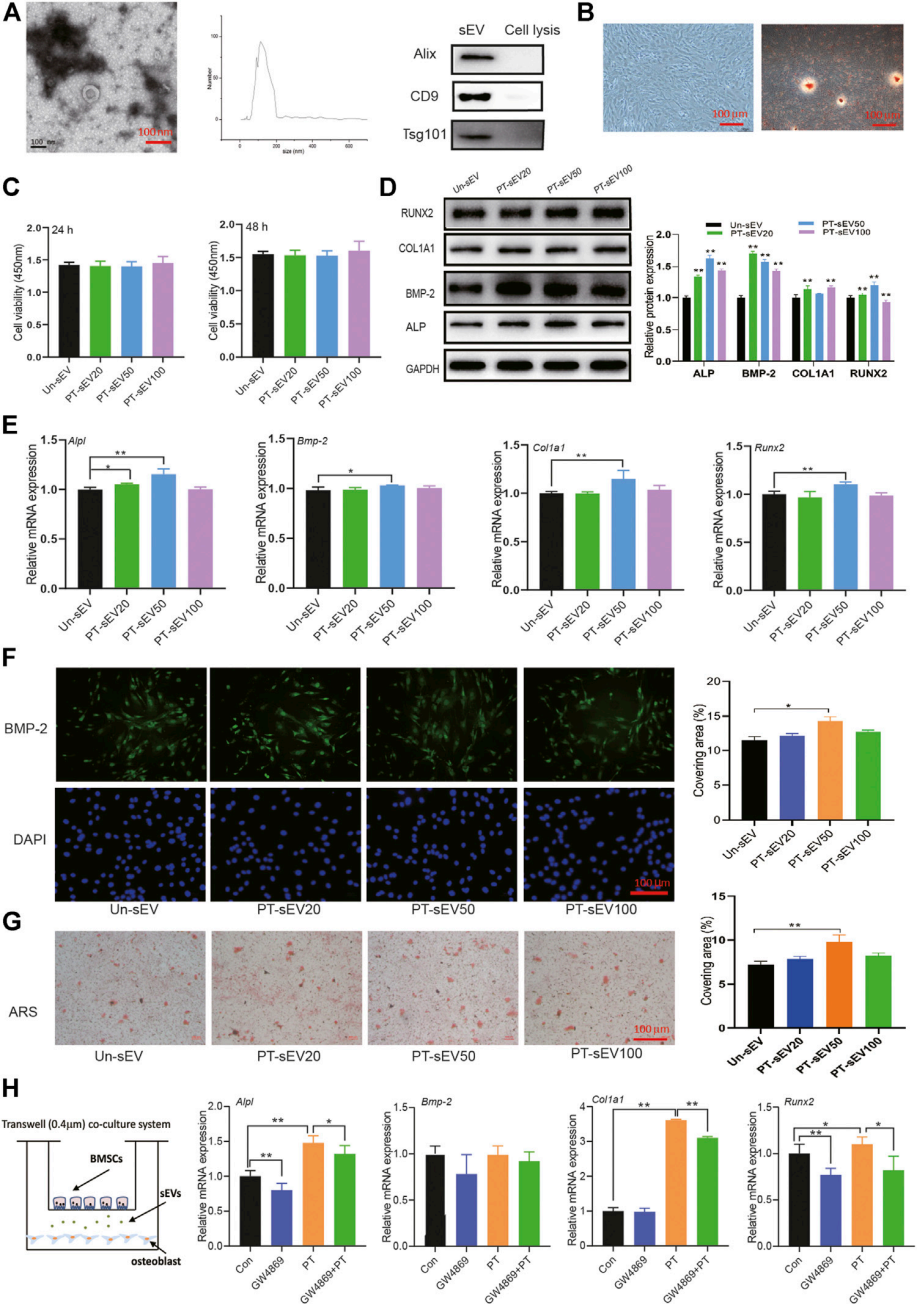
The osteogenic effects of PT-sEV on pre-osteoblasts. **(A)** The identification of sEVs, including scanning electron microscope and NTA results, scale bar: 100 nm; **(B)** The identification of pre-osteoblasts, including morphology and alizarin red s staining, scale bar: 100 μm; **(C)** The effect of PT-sEV on proliferation in pre-osteoblasts at 24 or 48 h, the raw data represented the mean ± SD, Un-sEV: sEVs dereived from untreated BMSCs; PT-sEV20: 20 μg/μl PT-sEV, PT-sEV50: 50 μg/μl PT-sEV, PT-sEV100: 100 μg/μl PT-sEV **(D, E)** The protein and mRNA expression of osteogenic differentiation related factor treated with PT-sEV, Un-sEV: sEVs dereived from untreated BMSCs; PT-sEV20: 20 μg/μl PT-sEV, PT-sEV50: 50 μg/μl PT-sEV, PT-sEV100: 100 μg/μl PT-sEV, the normalized data represented the mean ± SD; **(F)** The immunofluorescence results of BMP-2, scale bar: 100 μm; **(G)** The alizarin red s staining revealed the mineral nodules, scale bar: 100 μm; **(H)** The mRNA expression of osteogenic differentiation related factor under the co-culture system, the normalized data represented the mean ± SD. *p < 0.05, **p < 0.01, analyses were done twice and obtained comparable results.

The purified sEVs showed a typical cup-like appearance, with a double membrane structure and diameters ranging from 50 to 200 nm by NTA; the specific markers Alix, Tsg101, and CD9 were highly expressed in the sEVs (Figure 2A). The primary cells showed typical characteristics of pre-osteoblasts, including multi-tangle and elongated morphologies, and alizarin red S staining, which revealed various mineralized nodules (Figure 2B).

The effects of PT-sEV on pre-osteoblasts were evaluated by different assays. CCK8 results showed that different concentration of PT-sEV did not affect pre-osteoblasts proliferation after 24 h or 48 h; the protein and mRNA expression of the osteogenic related factors ALP, BMP-2, RUNX2, and COL1A1 was increased to different degrees, as were the results of immunofluorescence and alizarin S red staining Figures 2C–G).

We next co-cultured BMSCs and pre-osteoblasts with/without GW4869 to evaluate if PT stimulated osteogenic differentiation via sEVs. Compared with the Con group, the mRNA expression of *Alpl, Col1a1*, and *Runx2* was significantly increased in the PT group, while their expression was decreased in the combination PT + GW4869 group compared with the PT group (Figure 2H).

### PT-sEV Moderated Bone Mass and Bone Microstructure in OVX Rats

**FIGURE 3:**
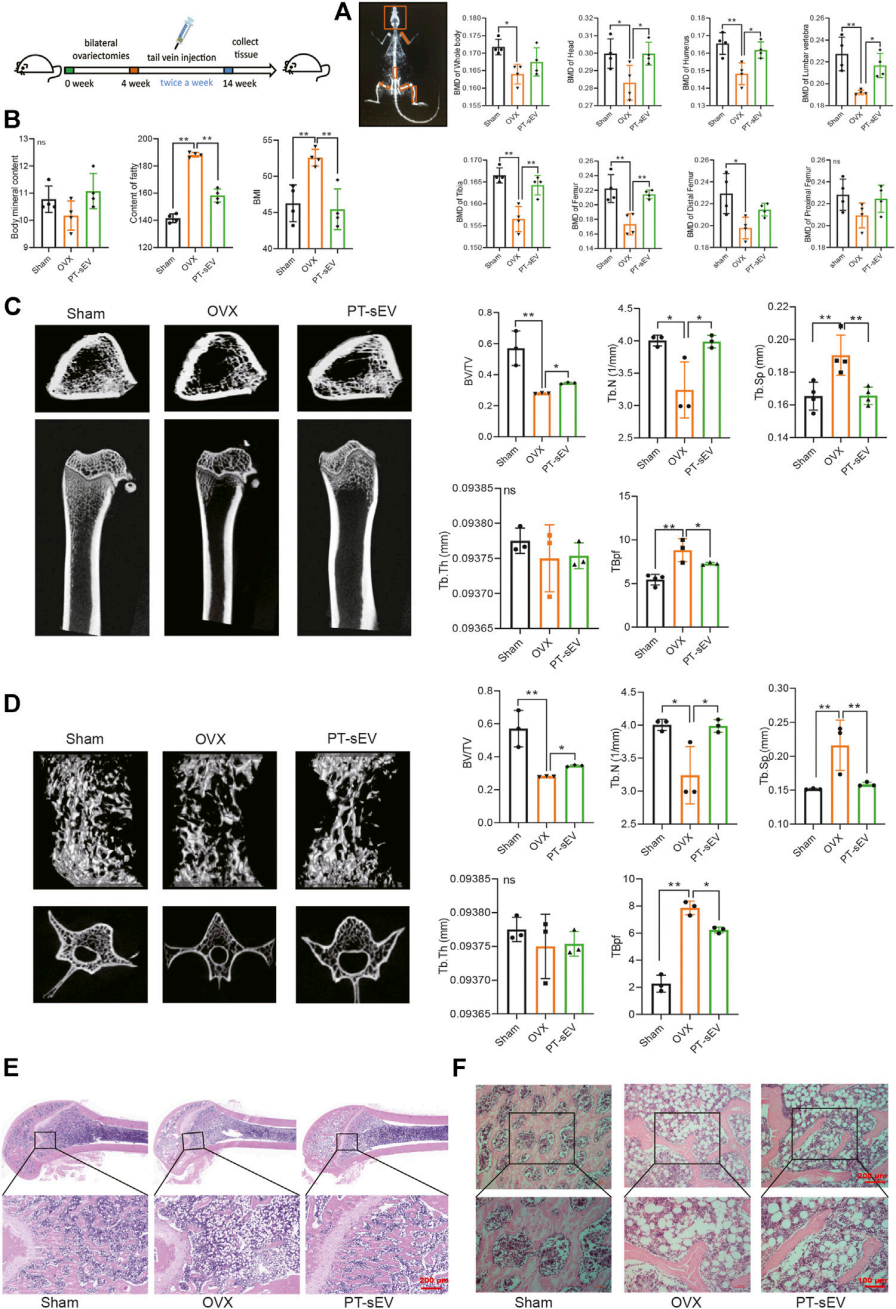
PT-sEV moderated bone mass and bone microstructure in OVX rats. **(A)** The BMD at different positions in different groups (*n* = 4), the raw data represented the replicate value; **(B)** The BMI, the content of fatty and content of BMC in different groups (*n* = 4), the raw data represented the replicate value; **(C)** The micro-CT results of femur (*n* = 4), the raw data represented the replicate value; **(D)** The micro-CT results of lumbar vertebrae (*n* = 3), the raw data represented the replicate value; **(E)** HE staining of femur, scale bar: 200 μm; **(F)** HE staining of lumbar vertebrae, scale bar: 200 μm or 100 μm *p < 0.05, **p < 0.01, ns: non-significant, analyses were done twice and obtained comparable results.

First, we analyzed the BMD at different positions in the different groups, which showed that the BMD of whole body, head, humerus, lumbar vertebrae, tibia, femur, distal femur, and proximal femur were lower in the OVX group compared with the sham group; additionally, PT-sEV significantly moderated the BMD of head, humerus, lumbar vertebrae, tibia, and femur in OVX rats (Figure 3A). Body mass index (BMI) and fatty content were increased, while the bone mineral content was decreased in the OVX group compared with the Sham group. With PT-sEV administration, fatty content and BMI decreased in the OVX rats (Figure 3B). We further analyzed the microstructure of the femur and lumbar vertebrae in OVX rats. The BV/TV and Tb.N were lower, and Tb. Sp and TBpf were higher than the Sham rats. PT-sEV treatment significantly moderated these effects (Figure 3C). Additionally, the altered trend of μCT parameters in the lumbar vertebrae was consistent with the femur (Figure 3D). HE staining showed that the trabecular bone appeared thinner, irregular, and discontinuous. There was also loss of reticular structure in the femur and lumbar vertebrae of the OVX group compared with the Sham group; PT-sEV administration helped restore the normal microstructure (Figures 3E,F).

### PT-sEV Stimulated the Expression of Bone Turnover Markers and Osteogenesis Related Factors in OVX Rats

**FIGURE 4:**
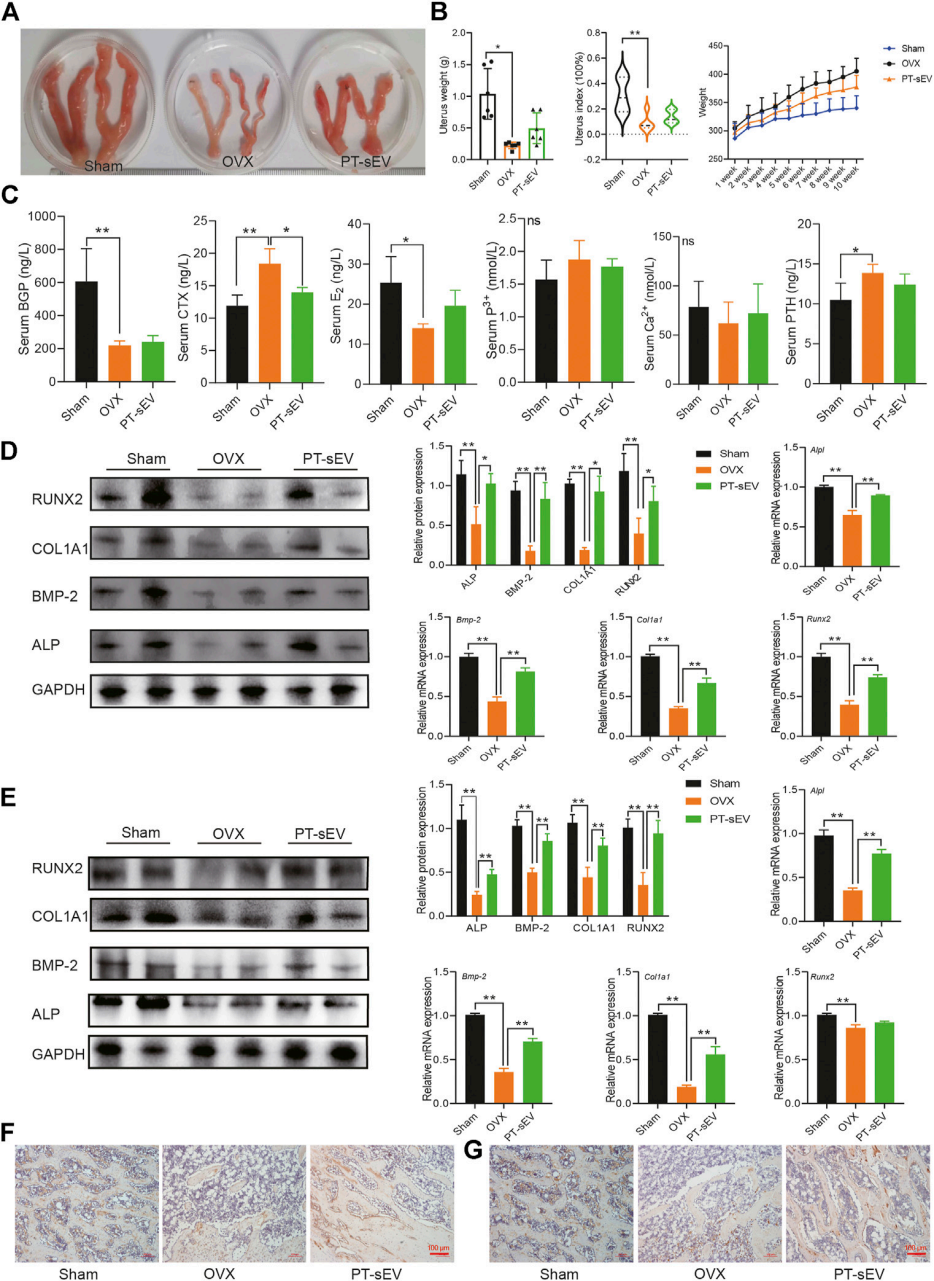
PT-sEV stimulated the expression of bone turnover markers and osteogenesis related factors in OVX rats. **(A)** The morphology of uterus in different group (*n* = 2); **(B)** The uterus weight, uterus index and body weight in different groups (*n* = 6), the raw data represented the replicate value; **(C)** The content of bone turnover biomarkers in serum, the raw data represented the replicate value; **(D)** The protein and mRNA expression of osteogenic related factor in femur, the normalized data represented the mean ± SD; **(E)** The protein and mRNA expression of osteogenic related factor in lumbar vertebrae, the normalized data represented the mean ± SD; **(F)** ICH results of COL1A1 in femur; **(G)** ICH results of COL1A1 inlumbar vertebrae, scale bar: 100 μm *p < 0.05, **p < 0.01, analyses were done twice and obtained comparable results.

Previous studies have indicated that various anti-osteoporosis drugs have estrogen-like effect; therefore, we first determined if PT-sEV induced any estrogen-like effect. To this end, we observed the morphology and weight of uteri from the different groups. The results showed that OVX rats had smaller uteri than the Sham group (Figure 4A). Although rats in the three groups had approximately initial weights, OVX rats gained more weight, followed by the PT-sEV group after different interventions for 10 weeks (Figure 4B).

Next, we analyzed serum levels of bone turnover markers. The results revealed that BGP and E2 levels were lower, while CTX was higher in the OVX group compared with the Sham group; PT-sEV attenuated CTX levels in the OVX group; and calcium and phosphorous levels were not significantly different among the different groups (Figure 4C). We then analyzed the expression of osteogenic differentiation related factors in the femur and lumbar vertebrae. The results showed that protein level of osteogenic differentiation factors were lower in the OVX group compared with the Sham group; PT-sEV administration significantly ameliorated these effects. The mRNA level of osteogenic differentiation factors showed similar trends as their corresponding protein (Figures 4D,E). Furthermore, IHC results showed reduced level and distribution of COL1A1 in the OVX group; PT-sEV significantly moderated these effects in OVX rats (Figures 4F,G).

### The miRNA Profile of PT-sEV

**FIGURE 5:**
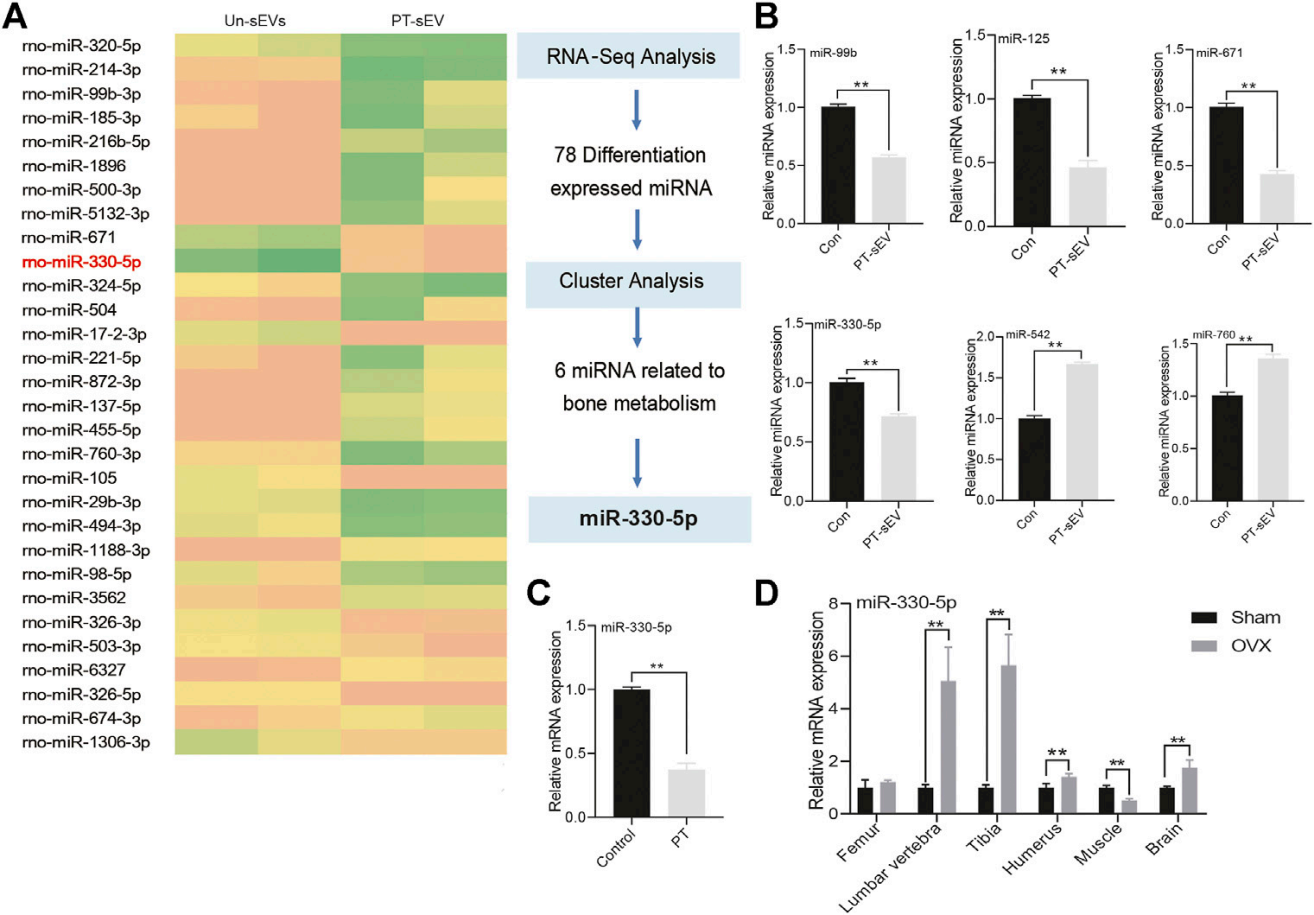
The miRNA profile of PT-sEV. **(A)** The heatmap of differentially expressed miRNA; **(B)** qPCR verified these differentially expressed miRNA in the different group, the normalized data represented the mean ± SD; **(C)** The miR-330–5p expression under the treatment of PT in BMSCs; **(D)** miR-330–5p expression at different tissues in OVX rats. The normalized data represented the mean ± SD. **p < 0.01, analyses were done twice and obtained comparable results.

To uncover the underlying mechanism for the increased osteogenesis in OVX rats, the miRNA profiles of PT-sEV were analyzed. The results showed that there were 201 differentially expressed miRNAs, including 120 upregulated and 81 downregulated miRNAs, such as miR-330-5p, miR-671 and miR-455-5p (Figure 5A). We used qPCR to verify the accuracy of RNA-Sequence, and these results confirmed the altered expression of miRNAs (Figure 5B). We also evaluated miR-330-5p expression in BMSCs following PT intervention (Figure 5C), as well miR-330-5p expression within different tissues in Sham and OVX rats. The results showed that miR-330-5p expression was significantly increased in the lumbar vertebrae, tibia, humerus and brain, but was significantly decreased in the muscle, but these data were not significantly different in OVX rats (Figure 5D).

### miR-330-5p Targeted Tenasin C to Attenuate Osteogenic Differentiation in BMSCs and Pre-Osteoblasts

**FIGURE 6:**
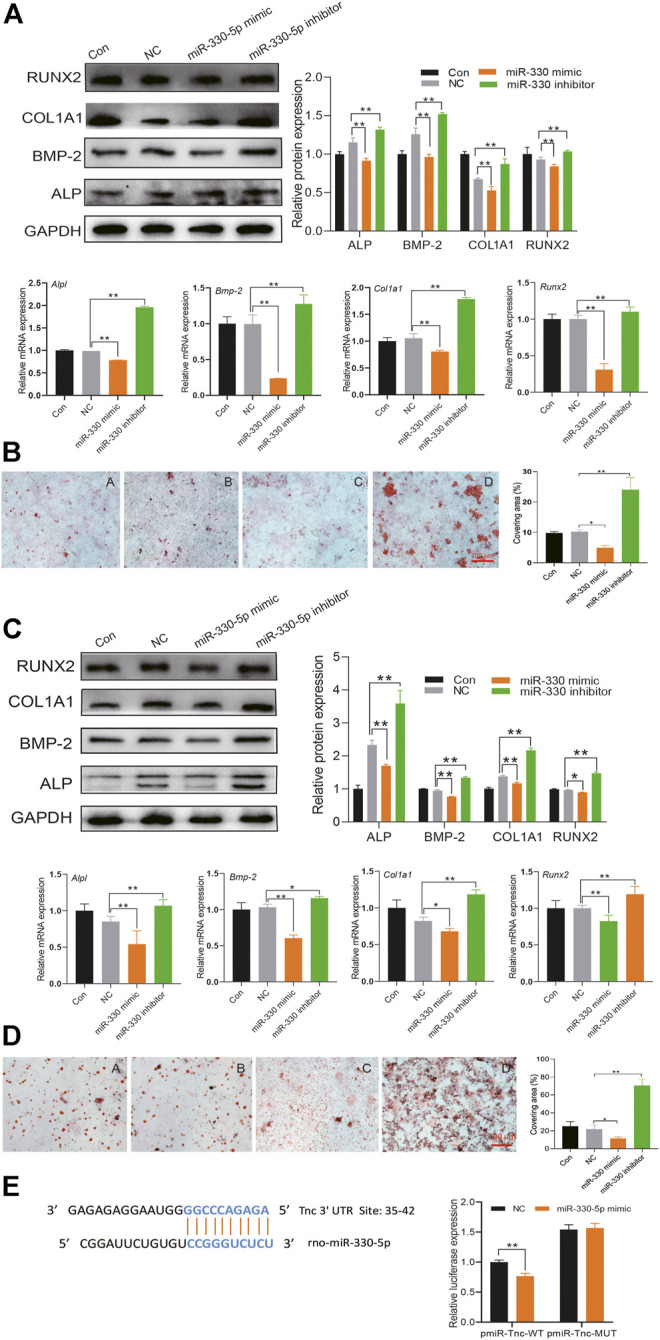
miR-330–5p targeted *Tnc* to attenuate osteogenic differentiation in BMSCs and pre-osteoblasts. **(A)** The protein and mRNA expression of osteogenic differentiation when overexpressing or inhibiting miR-330–5p in BMSCs, the normalized data represented the mean ± SD; **(B)** The representative pictures of alizarin red s staining in BMSCs, scale bar: 100 μm **(A)** Con **(B)** NC **(C)** miR-330–5p mimic **(D)** miR-330–5p Inhibitor; **(C)** the protein and mRNA expression of osteogenic differentiation when overexpressing or inhibiting miR-330–5p in pre-osteoblasts, the normalized data represented the mean ± SD; **(D)** The representative pictures of alizarin red s staining in pre-osteoblasts, scale bar: 100 μm **(A)** Con **(B)** NC **(C)** miR-330–5p mimic **(D)** miR-330–5p Inhibitor; **(E)** The dual-fluorescence reporter assay identify the affinity of Tnc and miR-330–5p. *p < 0.05, **p < 0.01, analyses were done twice and obtained comparable results.

To determine the effect of miR-330-5p on osteogenic differentiation, specific mimic and inhibitor were transfected into both BMSCs and pre-osteoblasts. The results showed that compared with the negative control group (NC), the protein and mRNA expression of osteogenic related factor was attenuated in the miR-330-5p mimic group, while the expression of these mRNAs and proteins were increased in the miR-330-5p inhibitor group (Figures 6A,C). Furthermore, alizarin red S staining showed that calcium deposits were increased in the miRNA inhibitor group, but decreased in the mimic group (Figures 6B,D). TargetScan and miRbase were used to predict target genes of miR-330-5p. Subsequently, a GFP reporter fusion gene was constructed, which showed that *Tnc* is a target gene of miR-330-5p. Compared with the NC group, miR-330-5p mimic significantly attenuated the fluorescence intensity of wild-type (WT) reporter but had no effect on the mutant reporter (Figure 6E).

### miR-330-5p Mediated Wnt Signaling to Regulate the Osteogenic Differentiation of Pre-osteoblasts

**FIGURE 7:**
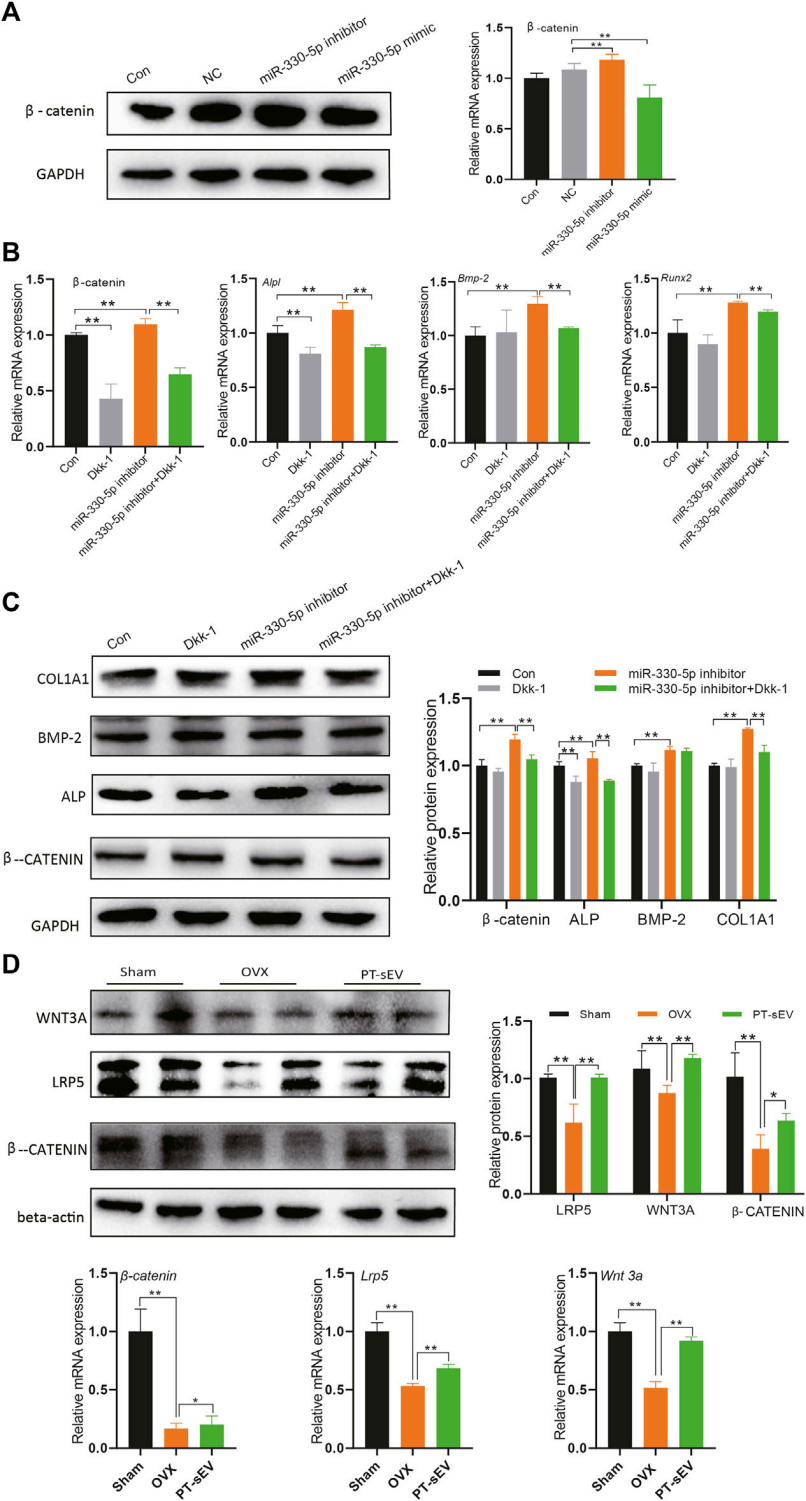
miR-330–5p mediated Wnt signaling to regulate the osteogenic differentiation of pre-osteoblasts. **(A)** The β-catenin expression when overexpressed or inhibited miR-330–5p, the normalized data represented the mean ± SD; **(B)** The rescue experiments showed the protein expression of osteogenic related factors and β-catenin, the normalized data represented the mean ± SD; **(C)** The rescue experiments showed the mRNA expression of osteogenic related factors and β-catenin, the normalized data represented the mean ± SD; **(D)** The Wnt signaling related factor expression in the femur, the normalized data represented the mean ± SD. *p < 0.05, **p < 0.01, analyses were done twice and obtained comparable results.

Previous research had indicated that Tnc is an inhibitor of Dkk-1, which acts as a traditional Wnt signaling inhibitor. In this study, we followed the published data and chose 10 μM Dickkopf-1 (Dkk-1) in the following experiment. Therefore, we hypothesized that miR-330-5p could regulate Wnt/β-catenin signaling. To further test this hypothesis, we evaluated β-catenin expression following treatment with miRNA mimic or inhibitor (Figure 7A). The results showed that β-catenin expression was increased with miR-330-5p overexpression, but was decreased by the miR-330-5p inhibitor in both pre-osteoblasts and BMSCs.

Further rescue experiment showed that miR-330-5p could block the effects of Dkk-1, while the osteogenic effects of miRNA mimic could be rescued by Dkk-1 in pre-osteoblasts (Figures 7B,C). Furthermore, we also examined the expression of Wnt related factors in the bone tissue. The results revealed that β-catenin, Wnt3a, and LRP5 were downregulated in OVX rats, PT-sEV treatment attenuated these effects in OVX rats (Figure 7D). We also analyzed the mRNA of *Tcf* and *Lef* (Supplementary Material S2) in the bone tissue, which are typical transcription factors for β-catenin expressed in the nucleus.

## Discussion

Osteoporosis is an aging related disease, which particularly affects postmenopausal women, who suffer high risks of fracture and morbidity. Currently, the existing therapeutic options for osteoporosis are flawed, so it is urgent to explore alternative agents that have fewer side effects. Several studies have shown that traditional Chinese medicines have beneficial effects for preventing and curing bone diseases (Abiramasundari et al., 2017). PT is a traditional Chinese medicine that has been used for many years to treat bone diseases. In this study, we showed that PT had no significant effects on the proliferation of BMSCs, but significantly promoted their osteogenic differentiation. Previous studies have shown that PT extracts can reverse glucocorticoid-induced spinal osteoporosis in rats via targeting osteoblastic and osteoclastic markers (An et al., 2016); PT extracts have also been shown to promote BMSCs proliferation and osteogenic differentiation by regulating Let-7f-5p and the TNFR2/PI3K/AKT signaling pathway (Liang et al., 2016). Therefore, it has been well-proven that the osteogenic effect of PT is closely related to BMSCs activity.

Recently, the beneficial effects of BMSCs for bone diseases have been attributed to paracrine effects induced by sEVs, which can modulate the bone microenvironment to maintain dynamic cellular homeostasis. In this study, we separated sEVs from PT-preconditioned or untreated BMSCs, and then demonstrated that PT-sEV could stimulate the proliferation, osteogenic differentiation, and mineral deposition of osteoblasts. Similarly, another recent study showed that sEVs derived from kartogenin-preconditioned MSCs could stimulate chondrogenesis (Shen et al., 2018). Dimethyloxaloylglycine-stimulated human BMSCs-derived sEVs also increased bone regeneration by promoting angiogenesis (Jing et al., 2020). Studies have indicated that several anti-osteoporosis drugs have estrogen-like effect; therefore, we wondered if there was any estrogen-like effect of PT-sEV. We observed that OVX rats had smaller and narrower uteri than rats of the Sham group, and PT-sEV did not significantly alter these phenotypes; additionally, serum E2 levels were not significantly different between the OVX and PT-sEV group. Therefore, we concluded that PT-sEV did not have estrogen-like effects.

We further evaluated osteogenesis in PT-sEV treated OVX rats. PT-sEV significantly enhanced BMD of the whole body, head, humerus, lumbar vertebrae, tibia, and femur in OVX rats. Liao et al. demonstrated that BMSCs-derived sEVs carrying miR-122-5p stimulated BMD during osteonecrosis of the femoral head (Liang et al., 2019). Femur and lumbar vertebrae more easily suffer fragility than other positions in osteoporosis; therefore, it is crucial to evaluate the altered bone mass and microstructure in these two sites. We used micro-CT and H and E staining to analyze bone quality and quantity in these two sites. OVX rats given PT-sEV showed moderated bone mass and bone microstructure, and elevated protein and mRNA levels of osteogenic differentiation related factors. Li et al. proved that sEVs secreted by BMSCs facilitated osteogenic differentiation (Liao et al., 2019). Liang et al. demonstrated that sEVs derived from MSCs enhance bone regeneration and angiogenesis in critical-sized calvarial defect rat models (Jing et al., 2020). Nevertheless, serum levels of bone turnover markers could reflect the status of bone metabolism to some degree, but there was no significant difference between the OVX and PT-sEV groups. Higher fat content and increased BMI are other characteristics of OVX rats (Nam et al., 2017; Li et al., 2021). In this study, PT-sEV diminished these phenotypes. In conclusion, PT-sEV not only stimulated osteogenic differentiation of osteoblasts, but also strengthened bone formation in OVX rats.

sEVs deliver cargo to receiving cells, which functions as a mean of communicating between cells. The different effects of sEVs depend on their cargo proteins, nucleic acids, and lipids. Previous studies have indicated that human adipose-derived stem cells altered the expression of exosomal miRNAs that promoted osteogenic differentiation (Sharma et al., 2017). Exosomal miR-128-3p from MSCs of aged rats regulates osteogenesis and bone fracture healing by targeting Smad5 (Yang et al., 2019). In this study, we analyzed the miRNA profile of PT preconditioned and untreated BMSCs, and found a series of miRNAs with altered expression, including miR-330–5p. miRNA gain and loss-of-function experiments showed that miR-330-5p attenuated osteogenic differentiation in pre-osteoblasts and BMSCs. Alizarin red S staining showed that miR-330-5p was negatively correlated with mineral nodules. Jin et al. demonstrated that silencing miR-330-5p could stimulate osteogenesis in BMSCs (Xu et al., 2020). Yoo et al. indicated that miR-330-5p was highly upregulated during BMSCs senescence (Jin et al., 2020), Therefore, miR-330-5p is a negative regulator of bone formation.

Further experimentation proved that the relevant target gene of miR-330-5p is Tnc, which is known to induce osteoblastic differentiation (Yoo et al., 2014) and protect against acute kidney injury by recruiting Wnt ligands. Tnc has also been shown to downregulate the Wnt inhibitor Dkk-1 in a neuroendocrine tumor model (Morgan et al., 2011). Therefore, we concluded that the function of miR-330-5p may involve the Wnt pathway. Therefore, we also detected the expression of β-catenin in the miR-330-5p gain and loss-of-function experiments, which showed that miR-330-5p positively regulates β-catenin expression. We then analyzed expressions of the Wnt pathway members LRP5, Wnt3a, and β-catenin in bone tissue, as well the mRNA expression of *Tcf* and *Lef.* The results showed that Wnt signaling was re-activated following PT-sEV treatment in OVX rats. Disrupting Wnt signaling in the osteoblastic lineage leads to bone formation defects and osteoporosis (Saupe et al., 2013). Huybrechts et al., 2020 reviewed the underlying genetic disorders with altered bone mass and found that all are involved in the canonical Wnt pathway (Jing et al., 2018). Further rescue experiments showed that miR-330-5p mimic could restore Wnt signaling, which was inactivated by Dkk-1. Thus, Wnt signaling positively regulated osteogenic differentiation of BMSCs and pre-osteoblasts.

## Conclusion

This study indicated that sEVs derived from PT-preconditioned BMSCs could stimulate osteogenesis in native BMSCs by delivering miR-330-5p, which targeted *Tnc* to modulate Wnt signaling. PT-sEV could become an innovative sEVs-based strategy of promoting osteogenic differentiation, which could be used in the future to prevent and cure osteoporosis.

## Data Availability

The data and materials used to support the findings of this experiment are available from the corresponding or first author upon reasonable request. The raw data has been deposited at: www.jianguoyun.com/p/DdsUHosQ0e_KCRju2YQE.
