# Supplementary material for: miR-330–5p in Small Extracellular Vesicles Derived From Plastrum testudinis-Preconditioned Bone Mesenchymal Stem Cells Attenuates Osteogenesis by Modulating Wnt/β-Catenin Signaling
Source: Front Mol Biosci. 2021 Aug 9;8:679345. doi: 10.3389/fmolb.2021.679345 (PMC8381775; doi:10.3389/fmolb.2021.679345)
Supplement: Supplementary file 1 [file Table1.docx]

**Table 1 The prime sequences**

| **Gene name** | **5’-3’ sequence** | |
| --- | --- | --- |
| ***Bmp-2*** | Forward:  Resverse: | GCCATCGAGGAACTTTCAGA  TGTTCCCGAAAAATCTGGAG |
| ***Alpl*** | Forward:  Resverse: | GACAAGAAGCCCTTCACAGC  ACTGGGCCTGGTAGTTGTTG |
| ***Col1a1*** | Forward:  Resverse: | ACGTCCTGGTGAAGTTGGTC  TCCAGCAATACCCTGAGGTC |
| ***Runx2*** | Forward:  Resverse: | AACAGCAGCAGCAGCAGCAG  GCACGGAGCACAGGAAGTTGG |
| ***β-catenin*** | Forward:  Resverse: | GAAAATGCTTGGGTCGCCAG  ATGGCAGGCTCGGTAATGTC |
| ***Tcf*** | Forward:  Resverse: | TACAGGGTTGCCACCAGAGT CTGTGCCTGCTGAGAGTGAA |
| ***Wnt3a*** | Forward:  Resverse: | TGGTGGTGGTGGTGGCAGAG  CACAGCCAAGGACCAGAGAAGAAC |
| ***Lrp5*** | Forward:  Resverse: | GGACATCGAGTTTGGTGGGA GTTGTTGTGGCGGTTCATGG |
| ***Lef*** | Forward:  Resverse: | CCCATCTTCACTTTCAGGGGAC  TAGCGTACACTCGGCTACGA |
| ***Gapdh*** | Forward: | GACATGCCGCCTGGAGAAAC |
|  | Resverse: | AGCCCAGGATGCCCTTTAGT |
