# Supplementary figures and images for: miR-330–5p in Small Extracellular Vesicles Derived From Plastrum testudinis-Preconditioned Bone Mesenchymal Stem Cells Attenuates Osteogenesis by Modulating Wnt/β-Catenin Signaling
Source: Front Mol Biosci. 2021 Aug 9;8:679345. doi: 10.3389/fmolb.2021.679345 (PMC8381775; doi:10.3389/fmolb.2021.679345)

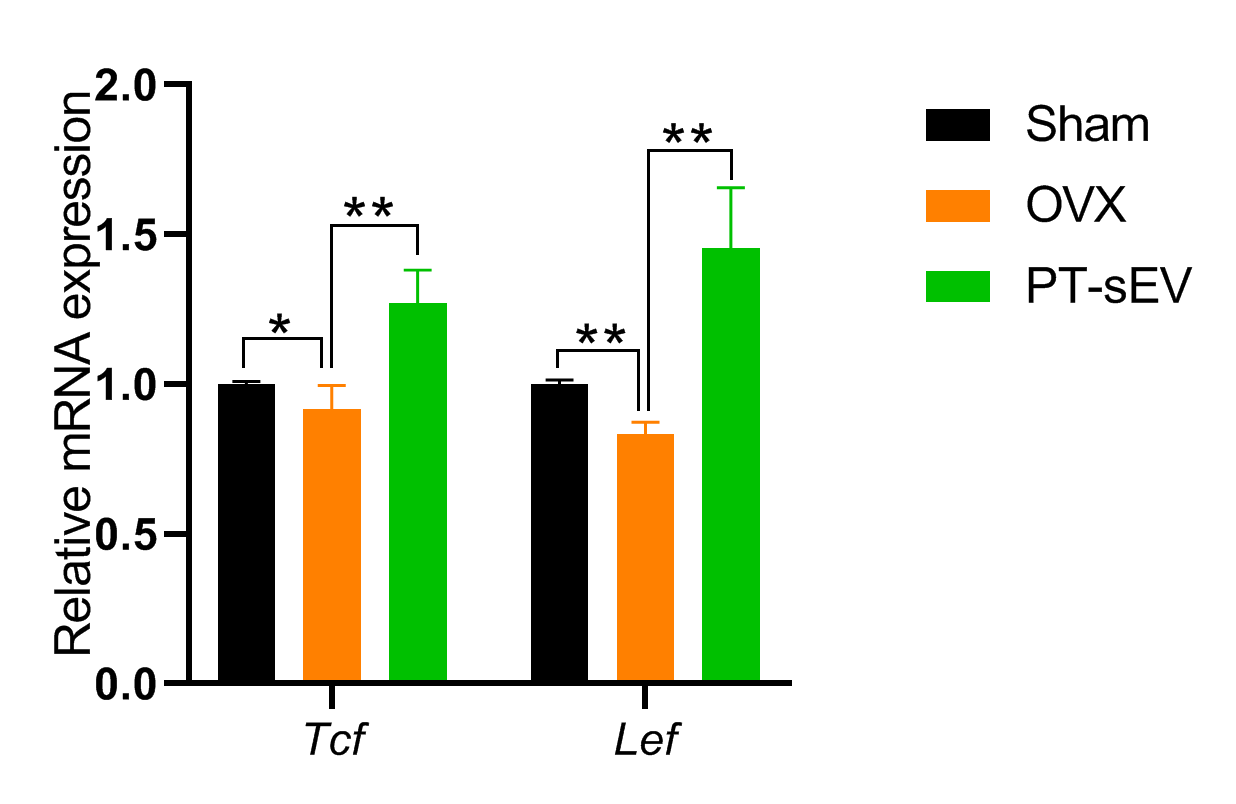

Supplement: Supplementary file 2 [file Image1.TIF]

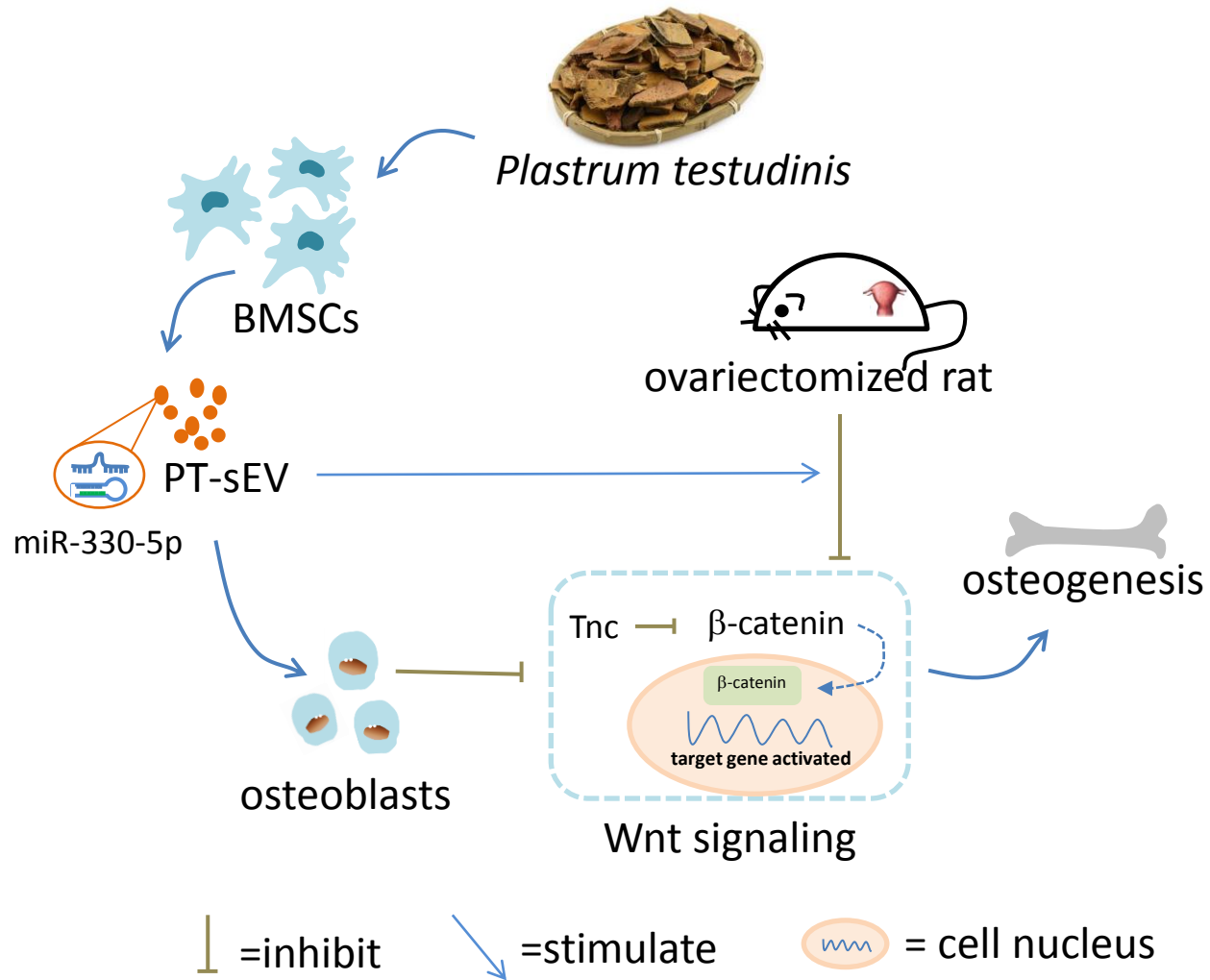

Supplement: Supplementary file 3 [file DataSheet1.PDF]
